# Comparative Evaluation of Aminoguanidine, Semicarbazide and Thiosemicarbazide Treatment for Methylglyoxal-Induced Neurological Toxicity in Experimental Models

**DOI:** 10.5812/ijpr-153322

**Published:** 2024-10-06

**Authors:** Noushin Nikray, Nikoo Abharian, Shahin Jafari Ashtiani, Farzad Kobarfard, Mehrdad Faizi

**Affiliations:** 1 Department of Pharmacology and Toxicology, School of Pharmacy, Shahid Beheshti University of Medical Sciences, Tehran, Iran; 2 Department of Medicinal Chemistry, School of Pharmacy, Shahid Beheshti University of Medical Sciences, Tehran, Iran

**Keywords:** Methylglyoxal, Advanced Glycation End Products (AGEs), Alzheimer’s Disease, Aminoguanidine, Semicarbazide, Thiosemicarbazide, Neurobehavior, Protein Carbonylation, Histopathology

## Abstract

**Background:**

Advanced glycation end products (AGEs) are complex compounds that play a critical role in neurological disorders, including the pathogenesis of Alzheimer's disease. Methylglyoxal (MG) is recognized as the primary precursor of AGEs. Methylglyoxal is produced endogenously and also introduced through dietary exposures.

**Objectives:**

This study aimed to investigate and compare the effects of aminoguanidine (AG), semicarbazide (SC), and thiosemicarbazide (TSC) on MG-induced neurological toxicity in rats.

**Methods:**

Male Wistar rats were exposed orally to MG, MG + AG, MG + SC, and MG + TSC for 70 days. Neurobehavioral, biochemical, and histopathological changes were evaluated.

**Results:**

The findings indicated that oral administration of MG for 70 days resulted in memory impairment and increased anxiety in neurobehavioral tests. Additionally, MG elevated protein carbonylation in brain tissues. Semicarbazide was found to prevent MG-induced memory problems, while both SC and AG reduced carbonyl content in brain tissues. Aminoguanidine and TSC were effective in alleviating anxiety induced by MG exposure. Histopathological analysis revealed that MG caused cell damage and neuronal necrosis in the hippocampus, particularly in the cornu ammonis 1 and 3 (CA1 and CA3) and AG, SC, and TSC improved neuronal survival specifically in the CA1 and DG areas.

**Conclusions:**

The data suggest that SC, AG, and TSC may offer neuroprotective effects against MG-induced neurobehavioral toxicity. Further studies are required to explore the mechanisms of action of these compounds.

## 1. Background

Alzheimer’s disease (AD), discovered in 1907, is one of the most significant neurodegenerative diseases (1, 2). The pathological results of AD include structural and functional damage, such as abnormal protein aggregation in neural tissue (2). Many therapeutic approaches in clinical trials have been suggested in recent decades. However, there is still no cure for AD, and more attention is focused on prevention and reducing the risk of AD (3). It has been demonstrated that glycation and advanced glycation end products (AGEs) are associated with chronic diseases, including AD, Parkinson’s disease (PD), and diabetes (4-8). Advanced glycation end products are heterogeneous, complex compounds formed either exogenously or endogenously through various mechanisms. These compounds are created by a non-enzymatic reaction (Maillard reaction) between carbonyl groups of reducing sugars (such as glucose and ribose) and free amine groups of proteins, nucleic acids, or lipids, followed by further rearrangements that make them resistant to metabolism (9). The formation of AGEs-crosslinked macromolecules is irreversible (10). These final products are resistant to proteases and become inactivated for normal structural, physiological, and biological functions (11).

The formation of AGEs and their interaction with receptors for advanced glycation end products (RAGE) exacerbate oxidative stress by activating the nuclear factor kappa B (NF-κB) pathway, which leads to the production of pro-inflammatory cytokines, including interleukin-1 and -6, and increases neuronal cell death and dysfunction (12-14). According to animal model studies, AGEs can accumulate in the brain, causing the glycation of tau protein and amyloid beta (Aβ), which may lead to the hyperphosphorylation of tau and the aggregation of Aβ. Both of these factors are critical processes in the pathogenesis of AD (15, 16).

Methylglyoxal (MG), a highly reactive dicarbonyl intermediate, is the primary precursor of AGEs (17) and acts as a neurotoxin in the progression of some metabolic and neurodegenerative diseases due to the formation of AGEs and oxidative damage (18). Although plasma glucose concentrations are approximately 25,000 times higher than MG levels, MG is up to 50,000 times more reactive than glucose in the glycation process and can quickly form adducts with cellular and extracellular proteins (19-21), lipids (22), and DNA (23). Methylglyoxal is formed endogenously in tissues and body fluids of humans and animals through various mechanisms, such as auto-oxidation of sugars, degradation of triosephosphates, metabolism of ketone bodies, and catabolism of threonine (24). The rate of MG formation is influenced by factors such as the type of organism, tissue, cell metabolism, and physiological or pathological conditions. Additionally, exogenous sources such as coffee, cigarette smoke, alcoholic drinks, and certain foods can increase MG formation (25, 26). 

It has been demonstrated that high serum levels of MG may accelerate cognitive impairment (27). Although mechanisms such as the glyoxalase system detoxify excess MG (28), impairments leading to increased concentrations of MG are nearly unavoidable in some diseases. While studies on the neurobehavioral toxicity of MG are limited (29, 30), there is substantial evidence linking elevated MG concentration to memory deterioration (7), diabetes complications (6, 31), and anxiety-like behaviors (32). Recent developments have focused on preventing the formation and accumulation of AGEs in vivo (33).

Aminoguanidine (AG) is a nucleophilic hydrazine that inhibits AGEs formation both in vivo and in vitro by forming adducts with carbonyl and dicarbonyl precursors such as MG and glyoxal (34). It has been demonstrated that AG can prevent hippocampal alterations in streptozotocin-induced dementia (35) and has neuroprotective effects against MG neurotoxicity (36). Given the structural similarity of AG, semicarbazide (SC), and thiosemicarbazide (TSC), we decided to investigate and compare the neuroprotective effects of these three compounds on MG-induced neurological toxicity using behavioral, biochemical, and histopathological experiments.

## 2. Objectives

The current study aimed to evaluate and compare the neuroprotective effects of AG, SC, and TSC on the neurobehavioral toxicity of MG using a series of behavioral tests, including the open field test, Rotarod test, grip strength test, hot plate test, and radial arm water maze test in rats. Additionally, we planned to measure the protein-carbonyl content in brain tissues to assess oxidative damage. Histopathological examinations were performed on three regions of the hippocampus (CA1, CA3, and DG) using hematoxylin-eosin (H&E) staining and cresyl violet staining to observe potential neuronal damage and survival.

## 3. Methods

### 3.1. Chemicals

Methylglyoxal, aminoguanidine bicarbonate, semicarbazide hydrochloride, thiosemicarbazide, dinitrophenylhydrazine (DNPH), bovine serum albumin (BSA), Coomassie blue G, trichloroacetic acid (TCA), and guanidine hydrochloride were all obtained from Sigma-Aldrich Co. (Germany). All other chemical compounds were sourced from local providers.

### 3.2. Animals and Treatments

**Figure 1.**
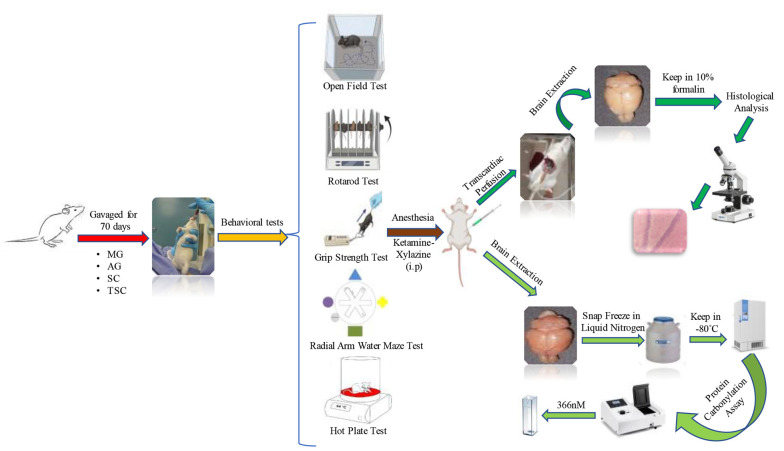
Schematic chart of the study design

Male Wistar rats (n = 80), weighing 180 - 200 g, were obtained from the Animal House of the School of Pharmacy, Shahid Beheshti University of Medical Sciences, Tehran, Iran, for experimental purposes. The rats were maintained under standard laboratory conditions (12 h light/dark cycles, controlled temperature of 22 ± 2°C, and relative humidity of approximately 50 ± 5%). They were housed in groups of 5 per cage and had free access to water and standard rodent food. The animals were randomly divided into eight groups: (1) control (Ctl), (2) MG, (3) MG-AG, (4) MG-SC, (5) MG-TSC, (6) AG, (7) SC, and (8) TSC. All compounds were dissolved in distilled water and administered orally (via gavage) to the animals daily for 10 weeks (37). The Ctl group received only the vehicle (distilled water) orally for 70 days. In the groups receiving MG, the animals were exposed to MG (120 mg/kg), and two hours later (to avoid drug interactions), AG (60 mg/kg), SC (60 mg/kg), or TSC (7.5 mg/kg) were administered to their respective groups. The doses were selected based on the literature (37) and pilot studies. The results from pilot studies confirmed that the doses of AG, SC, and TSC used were non-toxic. The study design and all experimental stages are outlined in Figure 1. All procedures were conducted in accordance with the ethical standards of the Institutional Animal Care and Use Committee (IACUC) of Shahid Beheshti University of Medical Sciences, with the approval code IR.SBMU.PHARMACY.REC.1400.315.

### 3.3. Behavioral Investigations 

#### 3.3.1. Open Field Test

Locomotor activity and anxiety-like behaviors were evaluated using the open field test. The open field apparatus was made from a transparent plexiglass box (60 cm³). Animals were placed individually inside the box for 10 minutes, and their movements were recorded using a digital camera (38). The time spent in the central zone (defined as the central area, which comprises half the total area of the arena) and the total distance moved by the rats were calculated using Ethovision tracking software (Noldus, the Netherlands).

#### 3.3.2. Rotarod Test

We investigated motor coordination and balance in the animals using the rotarod test. Each rat was placed individually on the rotarod device, equipped with an automatic timer (Borj Sanat Azma, Iran), with a 3-cm diameter rotating rod set at a speed of 5 rpm for a maximum duration of 60 seconds. The latency to fall off the rotarod within the 60-second period was recorded for each animal. The test was repeated three times for all subjects, and the average time spent on the rotarod was reported. Animals were allowed to rest for 10 minutes between trials (39).

#### 3.3.3. Grip Strength Test

The muscle strength in animals was measured using the grip strength test with a digital force meter (Borj Sanat Azma, Iran). Each rat was positioned horizontally, and when the forelegs touched the metal handle of the device, the rats were gently pulled backward by their tails until they released the handle. The peak grip strength (muscle strength) applied to the device was recorded automatically. The experiment was repeated three times, and the maximum force exerted was reported (40).

#### 3.3.4. Radial Arm Water Maze Test (RAWM)

We used the radial arm water maze to assess spatial memory and learning. The RAWM apparatus consisted of six arms placed in a swimming pool with water maintained at 25°C. During the training phase (the first two days), the platform was positioned at the end of a designated target arm, just two centimeters below the water surface. The target arm was different for each rat. Each day, animals underwent 15 trials to learn the location of the hidden platform using visual cues placed at the end of each arm. The rats were placed in one of the arms and given 1 minute to find the hidden platform. The latency to find the platform was measured during the first two days. On the third day (probe day), the platform was removed from the target arm, and we recorded the number of entries into the target arm, the duration spent in the target arm, and the latency to enter the target arm. Data collection was carried out using a digital camera positioned above the maze, and analysis was conducted using Ethovision software (Noldus, the Netherlands) (41, 42).

#### 3.3.5. Hot Plate Test

The evaluation of thermal pain in animals was conducted using the hot plate test. Each animal was placed individually on a hot plate device (Borj Sanat Azma, Iran) set at 55°C, and movement was restricted by a plexiglass cylinder. The latency to exhibit behavioral changes, such as hind paw licking or jumping, was recorded. Once these behaviors were observed, the animals were immediately removed from the hot plate. A cut-off time of 10 seconds was established for this experiment to prevent tissue damage (43).

### 3.4. Tissue Harvesting and Fixing

After 14 hours of fasting, all animals were anesthetized with an intraperitoneal injection of ketamine–xylazine (100 mg/kg and 10 mg/kg, respectively). Subsequently, a subset of rats from each group was randomly selected. For fresh frozen sample collection, brain tissues were immediately removed over ice, wrapped in aluminum foil, rapidly frozen in liquid nitrogen, and stored at -80°C until biochemical evaluation. 

The remaining animals underwent transcardiac perfusion with cold normal saline. Following perfusion, the brain tissues were carefully collected, fixed, and preserved in 10% formalin for histopathological analysis.

### 3.5. Histopathological Examination

The brain tissues fixed in 10% formalin were processed for paraffin embedding to facilitate histopathological assessments. Sections were cut to a thickness of 5 micrometers using a rotary microtome (Leica, Germany). Hematoxylin-eosin (H&E) and cresyl violet staining techniques were applied to the paraffin-embedded sections following standard protocols (44). The cornu ammonis 1 and 3 (CA1 and CA3) regions, as well as the dentate gyrus (DG) of the hippocampus, were examined using a light microscope (Nikon, Japan).

### 3.6. Estimation of Protein-carbonyl Content

The protein carbonyl level was determined following derivatization of the precipitated protein with 2,4-dinitrophenyl hydrazine (DNPH). In brief, 200 µL of DNPH solution (prepared in 2 N hydrogen chloride) was added to 1 mL of the homogenized brain tissue solution (with a protein concentration of 1 mg/mL) and incubated at room temperature for 1 hour, following vortex mixing. Blank samples were prepared by mixing 200 µL of 2 N hydrogen chloride with 1 mL of the protein sample, omitting the DNPH. Protein precipitation was carried out by adding 1.2 mL of 20% (w/v) trichloroacetic acid (TCA), and samples were incubated on ice for 15 minutes. The samples were then centrifuged at 10,000 × g for 5 minutes at 4°C, and the supernatants were discarded. The protein pellets were washed with 1 mL of an ethanol/ethyl acetate mixture (1: 1, v/v), vortexed, and centrifuged again at 10,000 × g for 5 minutes. This washing process was repeated three times to ensure removal of any free DNPH. 

After the final centrifugation, the supernatants were discarded, and the protein pellets were dried under a stream of nitrogen gas. The dried pellets were dissolved in 1 mL of 6M guanidine hydrochloride (prepared in 50 mM phosphate buffer, pH 2.3) by vortex mixing. Once the protein was fully dissolved, carbonyl content was measured by recording the absorbance at 366 nm against the blank. The concentration of total protein-bound carbonyl content was calculated using an extinction coefficient of 22,000 M^-1^ cm^-1^. The difference in absorbance was expressed as nanomoles of carbonyl content per milligram of protein (45).

### 3.7. Statistical Analysis

All data were presented as mean ± SEM. The normality of the data was assessed using the Shapiro-Wilk test, and parametric statistics were employed for data analysis. One- or two-way analysis of variance (ANOVA) with the corresponding post hoc LSD tests (Tukey’s or Bonferroni’s tests, respectively) were applied for comparisons between the studied groups. A value of P < 0.05 was considered statistically significant. All analyses were performed using GraphPad Prism software (version 8.0).

## 4. Results

The results of the evaluation and comparison between the studied groups are presented in two separate graphs for each experiment. The first graph compares the groups receiving MG (MG, MG-AG, MG-SC, and MG-TSC) to the Ctl group, while the second graph compares the groups receiving only AG, SC, or TSC to the Ctl group, in order to investigate the effects of these compounds on rats when used without MG.

### 4.1. Behavioral Investigations

**Figure 2.**
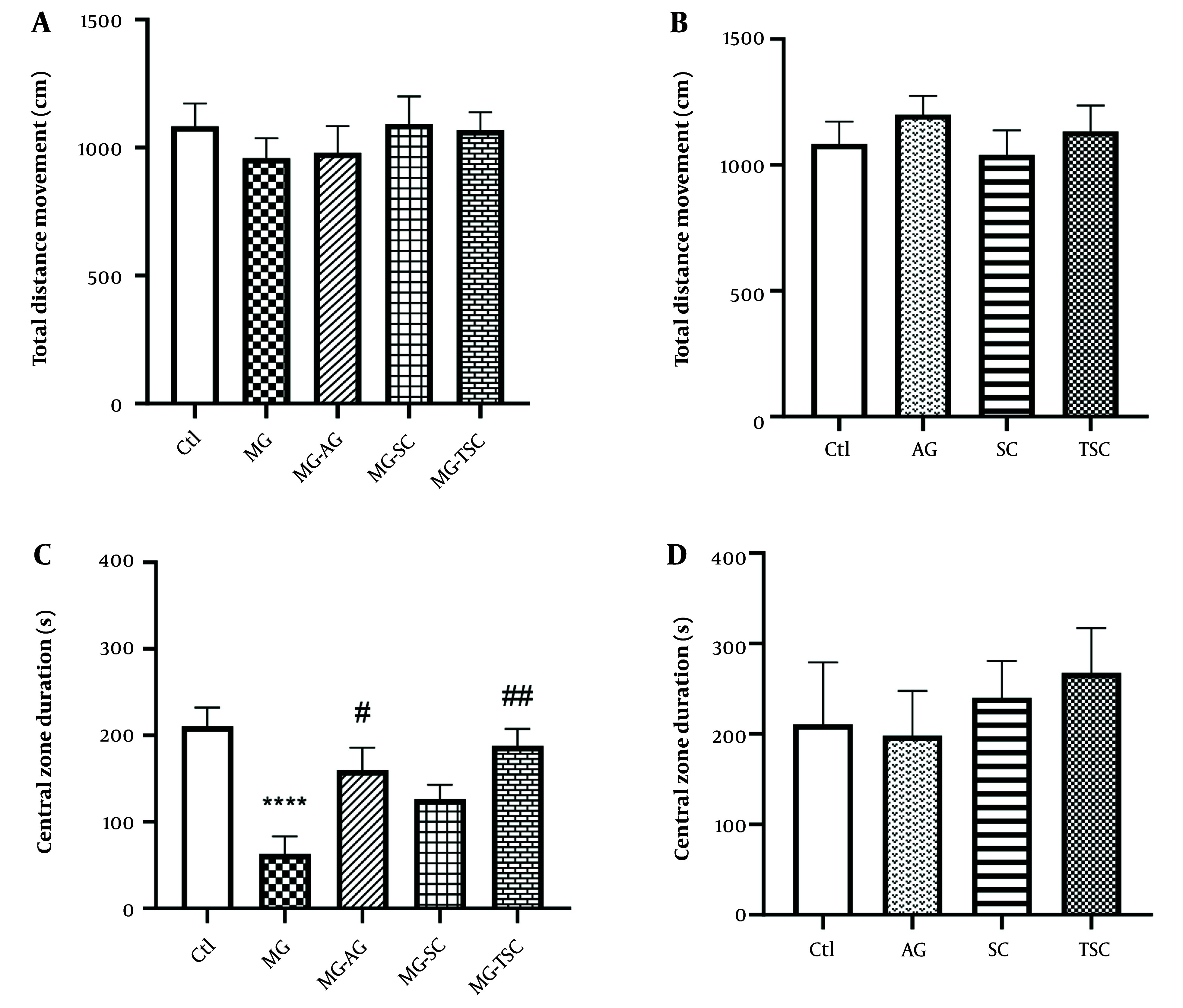
Effect of daily oral administration of MG, AG, SC, and TSC (for 70 days) on total distance movement (A, B); and central zone duration (C, D) by open field test. The results were analyzed by one-way ANOVA that followed by Tukey’s test. Data are expressed as mean ± SEM (n = 10 for each group). P < 0.05 is considered as significant level. **** P < 0.0001, # P < 0.05, ## P < 0.01. (*) As compared with the Ctl group; (#) as compared with the MG group. Abbreviations: Ctl, control; MG, methylglyoxal; MG-AG, methylglyoxal-aminoguanidine; MG-SC, methylglyoxal-semicarbazide; MG-TSC, methylglyoxal-thiosemicarbazide; AG, aminoguanidine; SC, semicarbazide; TSC, thiosemicarbazide.

As shown in Figure 2A, exposure to MG and treatment with AG, SC, and TSC for 70 days had no significant effect on the locomotor activity of animals in the open field test. We also evaluated the time spent in the central zone of the open field apparatus as an indicator of anxiety-like behaviors. The MG group spent significantly less time in the central zone compared to the Ctl group (P < 0.0001, Figure 2C). However, the time spent in the central zone was significantly increased in the MG-AG and MG-TSC groups compared to the MG group (P < 0.05 and P < 0.01, respectively; Figure 2C). In addition, the total distance moved and the time spent in the central zone did not show any significant changes in the animals that received only AG, SC, or TSC compared to the Ctl group (Figure 2B and D).

**Figure 3.**
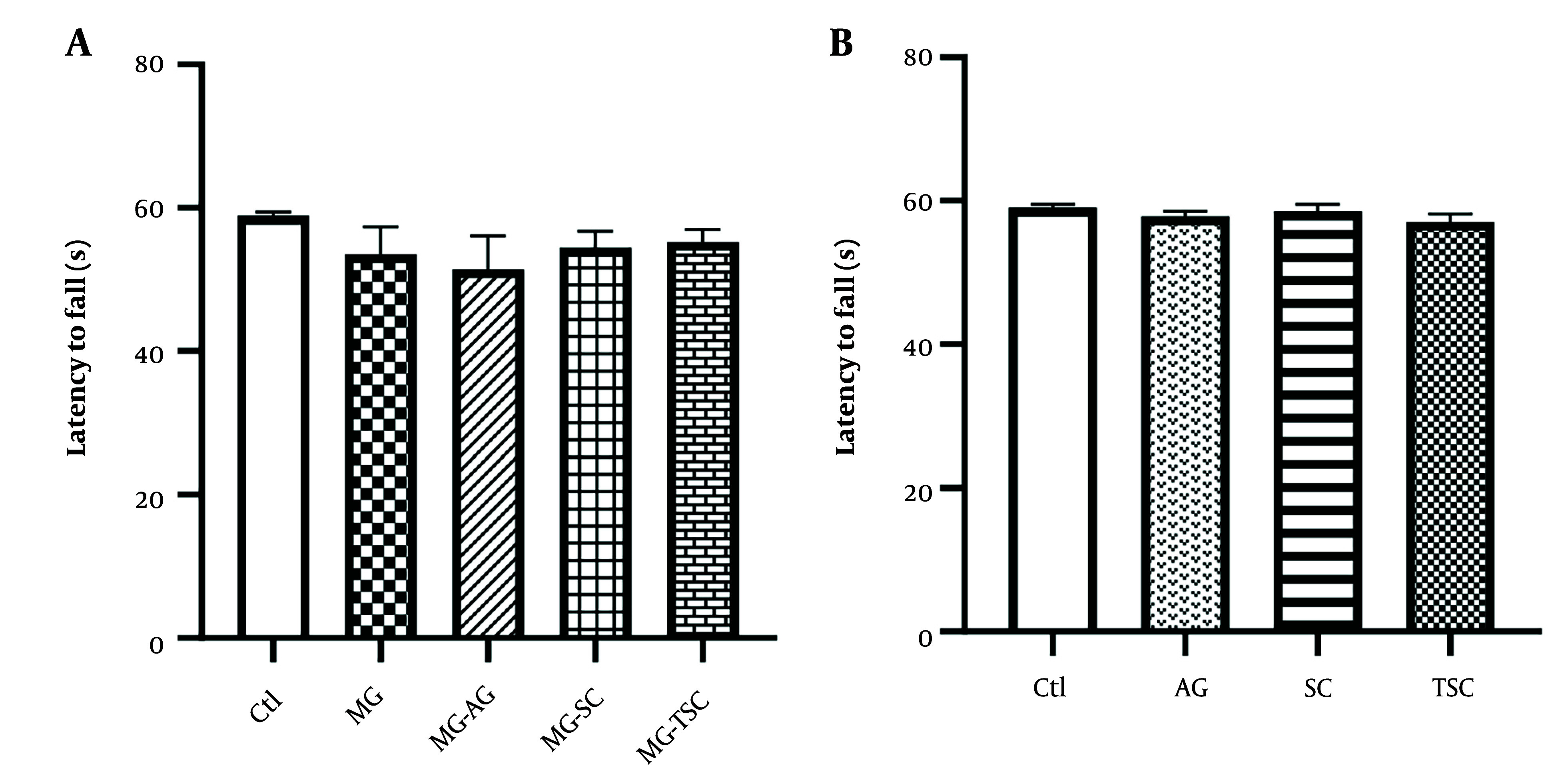
Effect of oral daily administration of MG, AG, SC, and TSC (for 70 days) on latency to fall by rotarod test. The results were analyzed by one-way ANOVA followed by Tukey’s test. Data are expressed as mean±SEM (n =10 for each group). P < 0.05 is considered as significant level. Abbreviations: Ctl, control; MG, methylglyoxal; MG-AG, methylglyoxal-aminoguanidine; MG-SC, methylglyoxal-semicarbazide; MG-TSC, methylglyoxal-thiosemicarbazide; AG, aminoguanidine; SC, semicarbazide; TSC, thiosemicarbazide.

We used the rotarod test to evaluate motor coordination in rats. As shown in Figure 3A and B, no significant changes were observed in latency to fall (seconds) between the studied groups.

**Figure 4.**
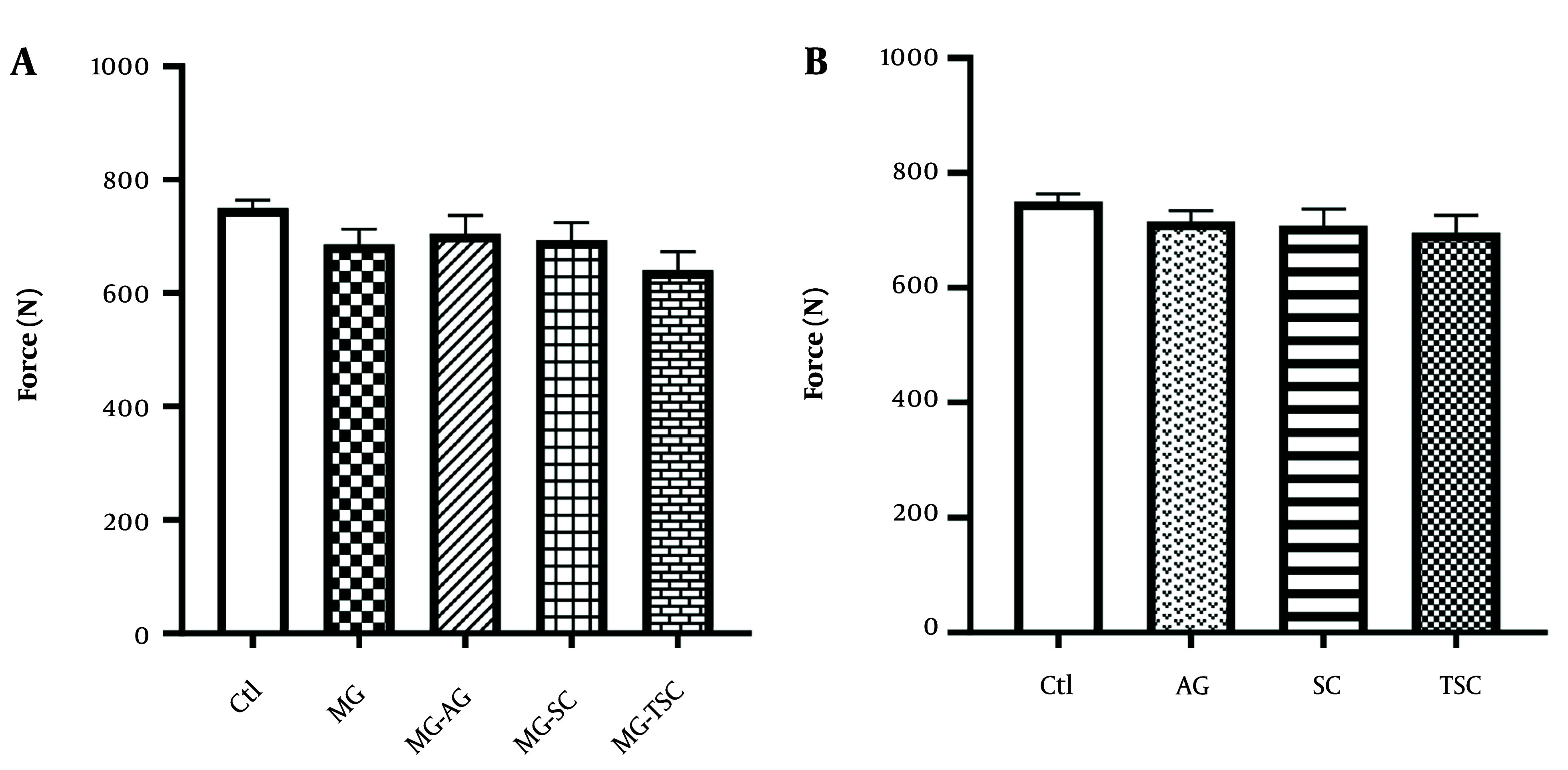
Effect of oral daily administration of MG, AG, SC, and TSC (for 70 days) on muscle strength by grip strength test. The results were analyzed by one-way ANOVA followed by Tukey’s test. Data are expressed as mean ± SEM (n =10 for each group). P < 0.05 is considered as significant level. Abbreviations: Ctl, control; MG, methylglyoxal; MG-AG, methylglyoxal-aminoguanidine; MG-SC, methylglyoxal-semicarbazide; MG-TSC, methylglyoxal-thiosemicarbazide; AG, aminoguanidine; SC, semicarbazide; TSC, thiosemicarbazide.

The grip strength test was conducted to assess muscle strength in the animals. As presented in Figure 4A and B, no significant differences in muscle strength were observed between the groups.

**Figure 5.**
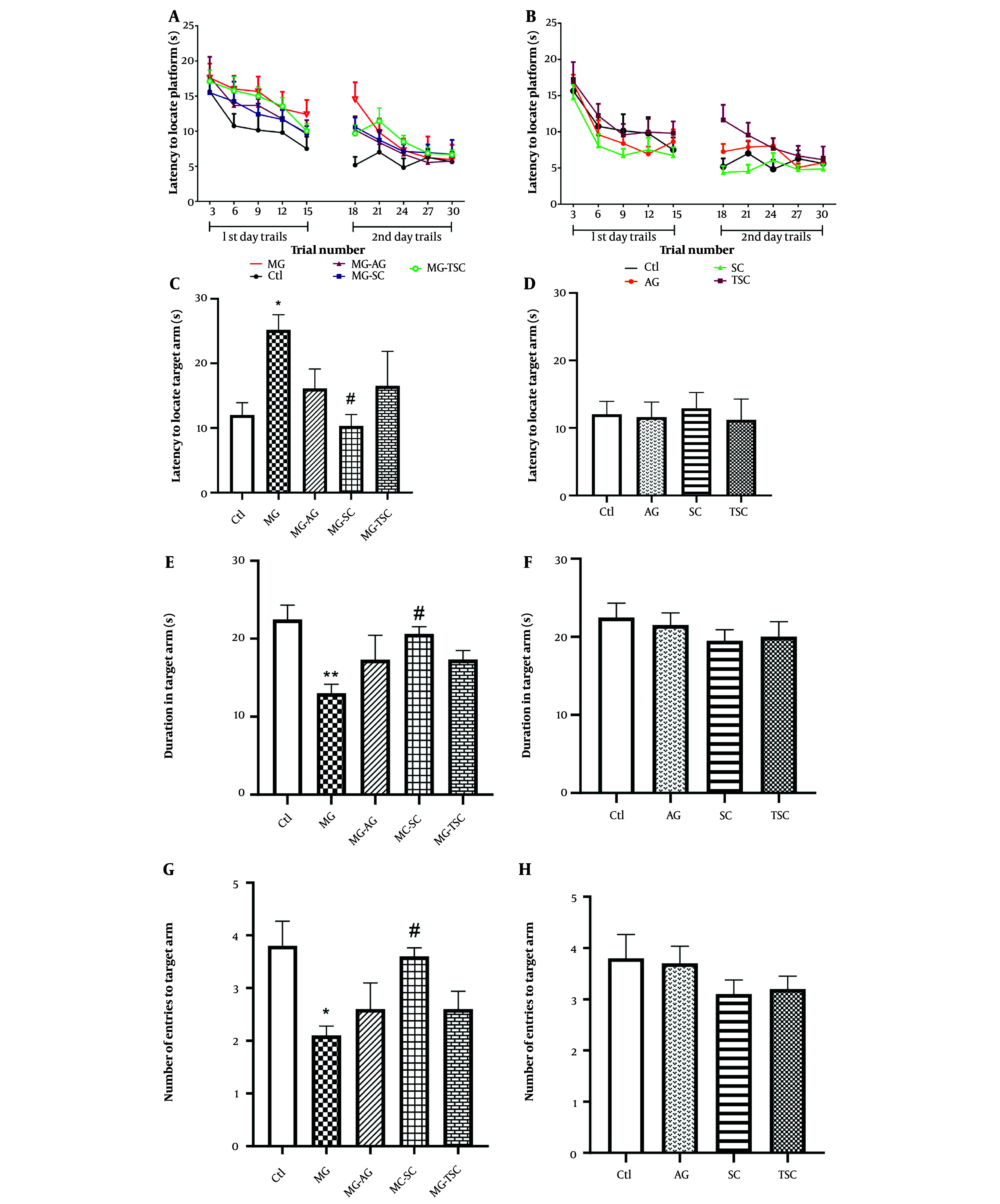
Effect of oral daily administration of MG, AG, SC and TSC (for 70 days) on latency to locate the platform (A, B); latency to locate the target arm (C, D); duration in target arm (E, F); and the number of entries to the target arm (G, H) by the radial arm water maze test. The results were analyzed by one- or two-way ANOVA followed by Tukey’s or Bonferroni’s tests. Data are reported as mean ± SEM (n = 10 for each group). P < 0.05 is considered as significant level. * P < 0.05, ** P < 0.01, # P < 0.05. (*) As compared with Ctl group; (#) as compared with MG group. Abbreviations: Ctl, control; MG, methylglyoxal; MG-AG, methylglyoxal-aminoguanidine; MG-SC, methylglyoxal-semicarbazide; MG-TSC, methylglyoxal-thiosemicarbazide; AG, aminoguanidine; SC, semicarbazide; TSC, thiosemicarbazide.

We investigated spatial learning and memory using the radial arm water maze test (RAWM). During the two training (trial) days, no significant difference in the latency to locate the hidden platform was observed in the MG-receiving groups compared to the Ctl group (effect of trial number, F (9, 81) = 25.61, P < 0.0001; effect of treatment, F (4, 36) = 2.13, P = 0.09; effect of interaction, F (36, 324) = 0.6, P = 0.96, Figure 5A). Additionally, the latencies to locate the hidden platform in the AG, SC, and TSC groups were not significantly different compared to the Ctl group (effect of trial number, F (9, 81) = 24.27, P < 0.0001; effect of treatment, F (3, 27) = 2.36, P = 0.09; effect of interaction, F (27, 243) = 0.75, P = 0.8, Figure 5B). 

On the probe (test) day, the latency to locate the target arm was significantly increased in the MG group compared to the Ctl group, and significantly decreased in the MG-SC group compared to the MG group (P < 0.05, Figure 5C). As shown in Figure 5E, the duration spent in the target arm (s) was significantly reduced in the MG group compared to the Ctl group (P < 0.01), while a significant increase in target arm duration was observed in the MG-SC group compared to the MG group (P < 0.05). Additionally, the number of entries to the target arm was significantly reduced in the MG group compared to the Ctl group and was significantly increased in the MG-SC group compared to the MG group (P < 0.05, Figure 5G). The AG, SC, and TSC groups did not show any significant differences compared to the Ctl group in all the probe tests (Figure 5D, F, and H).

**Figure 6.**
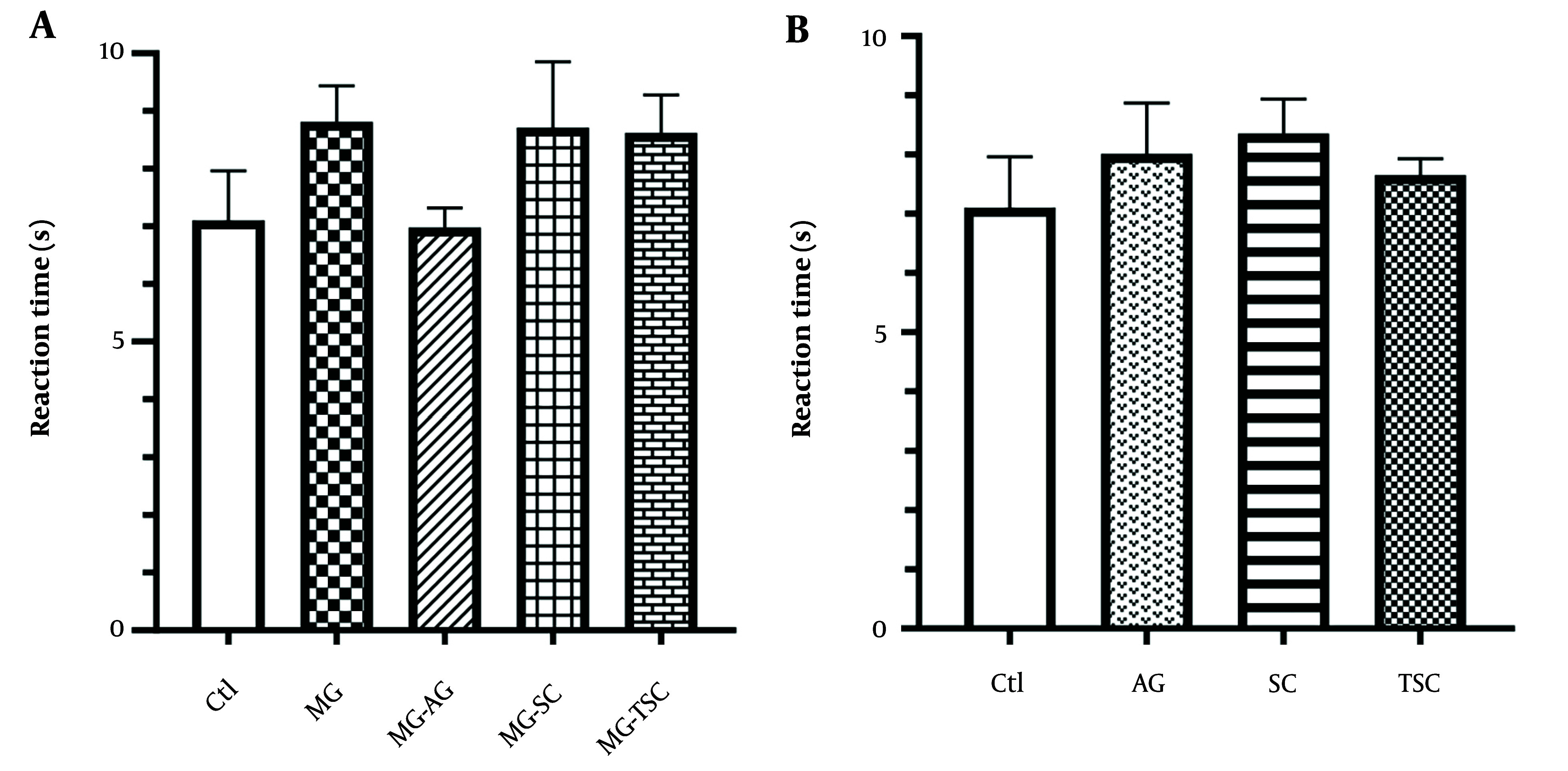
Effect of oral daily administration of MG, AG, SC and TSC (for 70 days) on pain by hot plate test. The results were analyzed by one-way ANOVA followed by Tukey’s test. Data are reported as mean ± SEM (n =10 for each group). P < 0.05 is considered as significant level. Abbreviations: Ctl, control, MG, methylglyoxal; MG-AG, methylglyoxal-aminoguanidine; MG-SC, methylglyoxal-semicarbazide; MG-TSC, methylglyoxal-thiosemicarbazide; AG, aminoguanidine; SC, semicarbazide; TSC, thiosemicarbazide.

The hot plate test was employed to assess the analgesic effect in the subjects. The reaction time showed no significant difference across the studied groups (Figure 6A and B).

### 4.2. Biochemical Assay

**Figure 7.**
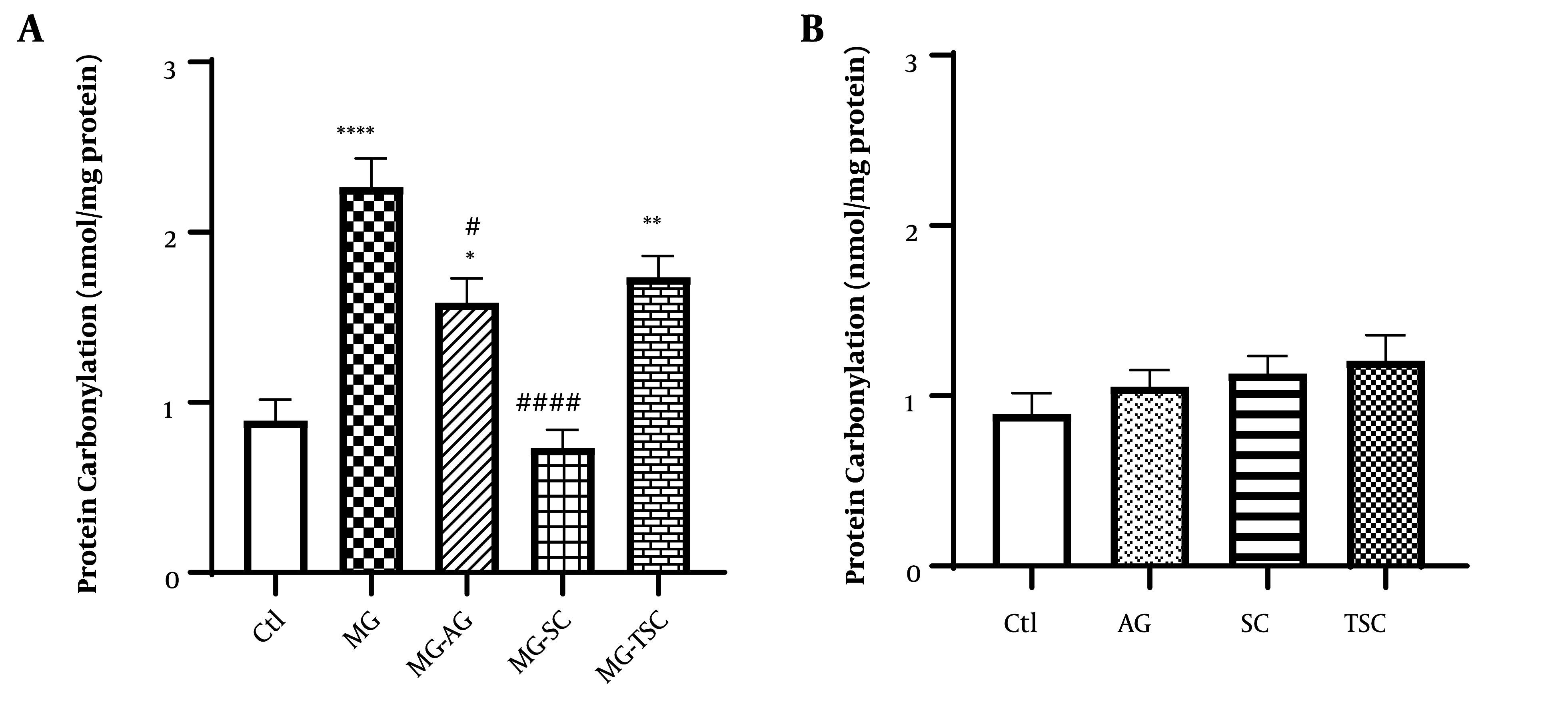
Effect of oral daily administration of MG, AG, SC and TSC (for 70 days) on protein-carbonyl content. The results were analyzed by one-way ANOVA followed by Tukey’s test. Data are reported as mean±SEM (n = 6 for each group). P < 0.05 is considered as significant level. * P < 0.05, ** P < 0.01, **** P < 0.0001, # P < 0.05, #### P < 0.0001. (*) As compared to the Ctl group; (#) as compared with MG group. Abbreviations: Ctl, control; MG, methylglyoxal; MG-AG, methylglyoxal-aminoguanidine; MG-SC, methylglyoxal-semicarbazide; MG-TSC, methylglyoxal-thiosemicarbazide; AG, aminoguanidine; SC, semicarbazide; TSC, thiosemicarbazide.

We measured the protein carbonyl content as an index of ROS-induced oxidation in proteins. As shown in Figure 7A, protein carbonylation was significantly increased in the MG, MG-AG, and MG-TSC groups compared to the Ctl group (P < 0.0001, P < 0.05, and P < 0.01, respectively). Additionally, there were significant decreases in protein carbonylation in the MG-SC and MG-AG groups compared to the MG group (P < 0.0001 and P < 0.05, respectively). Furthermore, no significant difference in protein carbonylation was observed between the groups that received AG, SC, or TSC when compared to the Ctl group (Figure 7B). 

### 4.3. Histopathological Analysis

**Figure 8.**
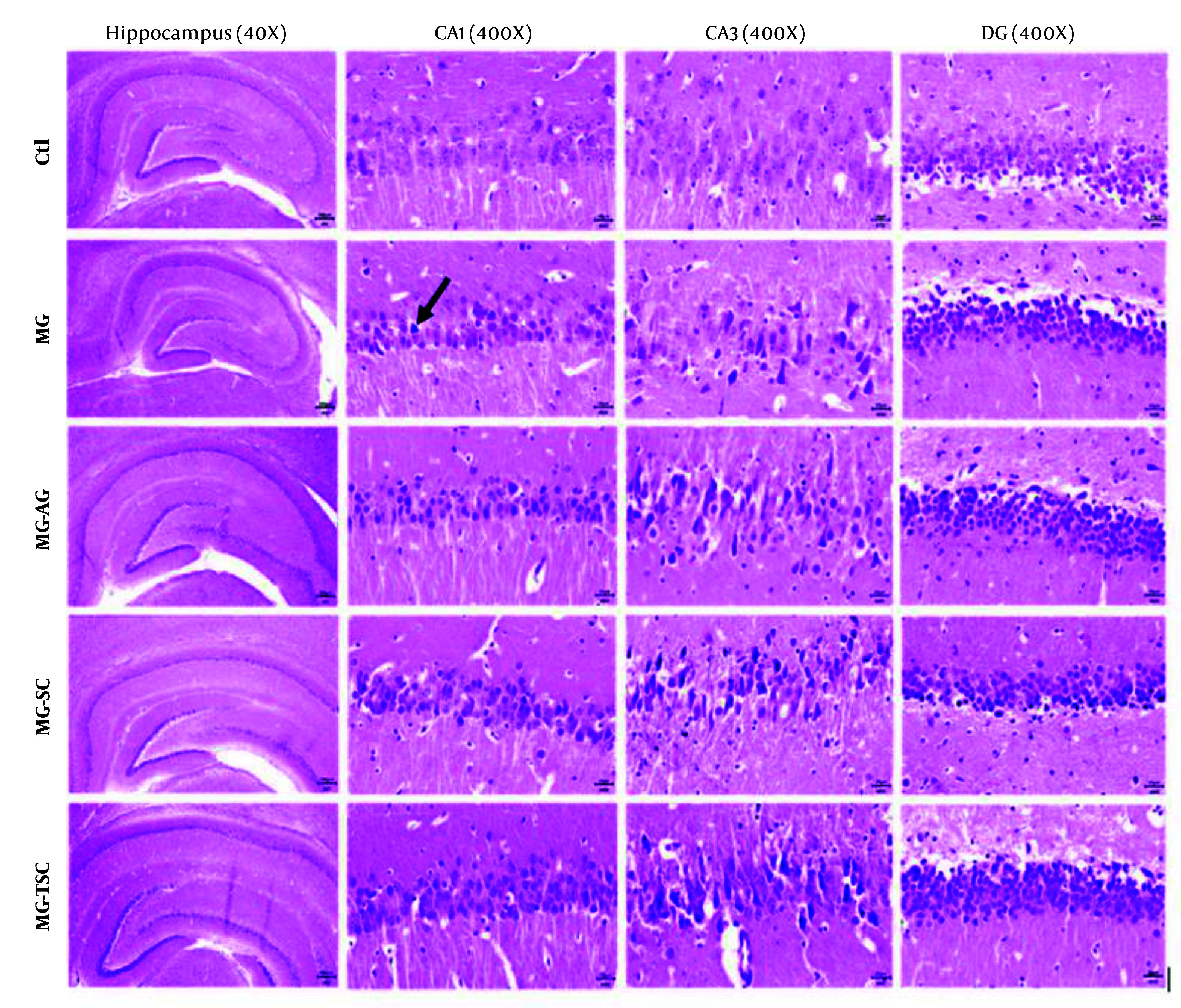
Histopathological analysis of the cornu ammonis 1 and 3 (CA1 and CA3) and dentate gyrus (DG) of the hippocampus in different treatment groups (H&E staining). The necrotic neurons are indicated by black arrows.

**Figure 9.**
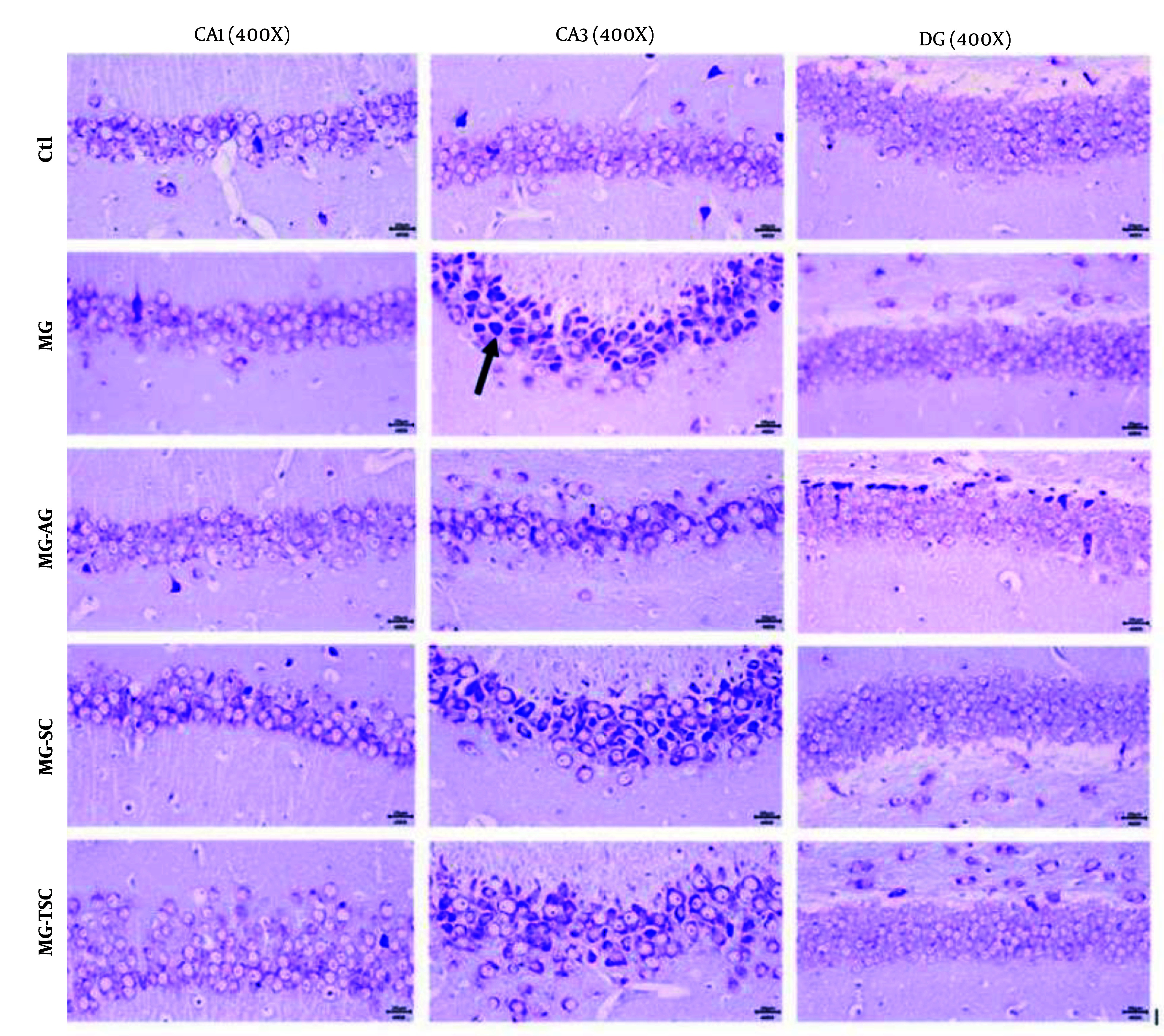
Histopathological analysis of the cornu ammonis 1 and 3 (CA1 and CA3) and dentate gyrus (DG) of the hippocampus in different treatment groups (Cresyl violet staining). The necrotic neurons are indicated by black arrows.

Histological investigation of the brain tissues was performed using light microscopy with hematoxylin-eosin (H&E) and cresyl violet staining. As shown in Figures 8 and 9, the histopathological examination of the hippocampal regions (CA1, CA3, and DG) revealed no evidence of neuronal loss or nuclear pyknosis in the CA1, CA3, and DG regions of the Ctl group. The most severe neuronal loss, necrosis, and disorganization of hippocampal neurons, especially in the CA1 and CA3 regions, were observed in the MG group. In the MG-AG group, neuronal structures in the CA1 region appeared relatively normal, with fewer necrotic neurons observed in the CA3 region. Neuronal necrosis and decomposition in CA3 were significantly reduced compared to the MG group. The neuronal structures in the CA1 region of the MG-SC group appeared more intact, and the CA3 region exhibited minimal signs of neuronal necrosis and decomposition compared to the MG group. The MG-TSC group demonstrated the best neuronal survival in the CA1 region, with relatively normal neuronal structures compared to the MG group. Although some necrotic neurons were observed in the CA3 region of the MG-TSC group, the extent of neuronal necrosis and decomposition was significantly less than MG group. The DG area showed the best organization and the highest number of normal neurons among all the treated groups.

## 5. Discussion

Methylglyoxal, a highly potent dicarbonyl compound, is the most important precursor of AGEs. One of the primary sources of MG and AGEs is food, particularly Western diets, and alcoholic beverages; however, they can also be produced endogenously as a by-product of the glycolysis pathway (46). Previous studies have shown that MG and AGEs contribute to cognitive impairments (27, 47, 48). The role of AG in improving diabetes complications caused by increased serum AGE levels has been demonstrated in various studies (49-51). This study aimed to evaluate the neurobehavioral toxicity of MG in male Wistar rats. Additionally, we investigated the neuroprotective effects of AG, SC, and TSC, due to their structural similarity, on MG neurotoxicity using a battery of behavioral tests, followed by biochemical and histopathological assessments.

Based on the results, long-term treatment (70 days) with MG induced anxiety-like behaviors by reducing the time spent in the central zone of the open field apparatus. Furthermore, anxiety-like behavior was significantly reduced in the MG-AG and MG-TSC groups compared to the MG group. These findings suggest that AG and TSC may have an anxiolytic effect on MG-induced neurotoxicity. Patil et al. treated Sprague-Dawley rats with oral MG (50 mg/kg/day) or in combination with AG (1 g/L/day, through drinking water) for 45 days. Their results from the elevated plus maze test showed that anxiety developed in rats after MG treatment, as evidenced by the reduction in the number of entries and distance traveled in the open arms. They reported that AG administration for 45 days reduced anxiety behavior compared to the MG group (51).

Methylglyoxal can induce oxidative stress by increasing AGE formation (52), inactivating antioxidant enzymes (53), and activating inducible nitric oxide synthase (iNOS) (54). It has been shown that AG can reduce anxiety by interfering with the iNOS-cGMP pathway. iNOS can influence anxiety through nitric oxide production, leading to an increase in cyclic guanosine monophosphate (cGMP). This secondary messenger enhances neuronal communication, contributing to increased anxiety. Additionally, AG is able to inhibit guanylyl cyclase, an essential enzyme involved in cGMP production, thereby reducing nitric oxide synthesis and cGMP formation, which results in an anxiolytic effect (55, 56).

We investigated learning and memory impairment using the radial arm water maze test. In the MG group, there was an increase in the latency to locate the target arm on the probe day. Additionally, the number of entries into the target arm and the duration spent in the target arm were both reduced in the MG group.

Furthermore, the latency to locate the target arm was decreased in the MG-SC group, and the number of entries to the target arm and the duration spent in the target arm showed an increase compared to the MG group. These findings suggest that SC may prevent memory impairment induced by MG treatment. Previous research has indicated that MG, as a major precursor of AGEs, is linked to cognitive decline (20, 57, 58). Methylglyoxal reacts with the free amine groups of amino acids (59), leading to AGE formation and oxidative damage to proteins (60). Long-term oral MG treatment elevates serum MG levels (61). Methylglyoxal detoxification primarily occurs through glyoxalase 1 (GLO1) and glyoxalase 2 (GLO2). Glyoxalase 1 converts methylglyoxal-glutathione hemithioacetal into S-(D)-lactoylglutathione, which is subsequently hydrolyzed by GLO2 into D-lactate and glutathione (62). 

Pucci et al. found that MG treatment decreases GLO1 activity, leading to MG accumulation in the hippocampus (61). As a result, RAGE expression increases, and the AGE-RAGE axis is activated (7). The AGE-RAGE axis plays a crucial role in cognitive impairment by activating intracellular signaling pathways, such as nuclear factor-κB (NF-κB), which lead to the production of pro-inflammatory cytokines like IL-1 and IL-6 (63, 64). Additionally, it increases reactive oxygen species (ROS) production, causing inflammation in neural tissues (65-67).

High intracerebroventricular (ICV) injection of MG (3 µmol/µL/day) administered over six days led to a decrease in the recognition index in the object recognition test but had no effect on learning and memory assessed by the open field and Y-maze tests (67). In another study, a high ICV injection of MG (3 µM/µL) impaired cognitive functions, including both short- and long-term memory, as well as short-term spatial memory, as evaluated by the novel object recognition and Y-maze tests (29). Treatment of mice with MG (100 mg/kg, orally) for four weeks also caused working memory impairment as shown by the Y-maze test (61). Long-term treatment with MG (0.5% in drinking water) in Sprague-Dawley rats did not cause significant cognitive impairment in spatial and working memory, though the serum MG level increased (68). This body of evidence demonstrates that whether or not cognitive impairment occurs depends on the specific MG study protocol employed.

In another part of our study, we measured the protein-carbonyl content in the brain tissues of the studied animals. The binding of reactive aldehydes to amino acid side chains, known as protein carbonylation, is one of the major irreversible oxidative protein alteration markers related to various chronic diseases such as AD and Parkinson's disease (69-72). Carbonyl stress accumulates reactive carbonyl species that interact with nucleophilic substrates, inducing biomolecular malfunction and altering crucial cellular proteins (24, 73). This leads to inflammation and autoimmune reactions (74). Based on the results, MG treatment for 70 days increased protein-carbonyl content in the brain tissues of the studied animals. We observed a significant increase in protein carbonylation in the MG, MG-AG, and MG-TSC groups compared to the control group. Furthermore, the MG-SC and MG-AG groups showed a decrease in protein-carbonyl content compared to the MG group. Preventing carbonyl stress is a new therapeutic approach for reducing AGEs formation by inhibiting the formation of Schiff bases and Amadori adducts (75, 76). Colzani et al. (2016) evaluated and compared the activity of AG toward four reactive carbonyl species using high-resolution mass spectrometry (HRMS) in vitro, and reported that AG's quenching reactivity depends on the type of reactive carbonyl species (RCS). AG inhibits protein carbonylation by binding to carbonyl or dicarbonyl RCS, including MG and glyoxal (77). These findings suggest that SC and AG may serve as practical agents for reducing MG-induced protein carbonylation, possibly by trapping dicarbonyl groups. Further research is needed in this area.

It has been demonstrated that cognitive functions such as memory, learning, and anxiety are closely related to hippocampal function (51). In this study, we examined the effects of MG, AG, SC, and TSC treatment on the CA1, CA3, and DG regions of the hippocampus using hematoxylin-eosin (H&E) staining and cresyl violet staining. According to the histological results, neuronal necrosis was most prominent in the CA3 region across all treatment groups. The MG group exhibited extensive neuronal loss and nuclear pyknosis, particularly in the CA1 and CA3 regions. In contrast, the other groups (MG-AG, MG-SC, and MG-TSC) demonstrated improved neuronal survival and reduced necrosis, with the MG-TSC group showing the best outcomes, particularly in the CA1 and DG areas. It has been shown that the central nervous system is sensitive to the toxic effects of MG (30). In one study, oral administration of MG (50 mg/kg/day) for 45 days led to mild neuronal degeneration in the CA1 region of the hippocampus, while co-treatment with AG (1 g/L/day) significantly reduced degenerative changes compared to the MG group (51). Another study exposed Sprague-Dawley male rats to MG (0.5% in drinking water) for 12 months, and their histological results indicated no significant differences in cellular morphology or apoptosis compared to the control group (68).

Overall, our findings support the idea that long-term MG exposure can mediate behavioral, biochemical, and histopathological changes in rat brains, affecting memory, anxiety, protein-carbonyl content, and cellular degeneration. Additionally, our results suggest that AG, SC, and TSC hold promise for further investigation as potential neuroprotective agents against MG-induced neurobehavioral toxicity.

### 5.1. Conclusions

Methylglyoxal is known as a highly reactive by-product of glucose metabolism and serves as a major precursor of AGEs, playing a critical role in the development of neurodegenerative diseases, including AD and Parkinson's disease. In addition to its endogenous production, MG is also introduced into the body through exogenous sources such as food and cigarette smoke. This study aimed to examine the neurobehavioral toxicity of MG, as well as evaluate and compare the neuroprotective effects of AG, SC, and TSC on MG-induced neurotoxicity using neurobehavioral, biochemical, and histopathological assessments.

We observed that oral administration of MG impaired memory and increased anxiety-like behaviors in animals. Additionally, MG treatment led to increased protein carbonylation in brain tissue, a marker of oxidative stress. SC was effective in preventing memory impairment, while both SC and AG reduced protein carbonylation in the brain tissue. Aminoguanidine and TSC also decreased anxiety-like behaviors. Furthermore, MG exposure caused neurodegenerative changes in the hippocampus, specifically in the CA1 and CA3 regions and AG, SC, and TSC improved neuronal survival particularly in the CA1 and DG areas.

According to the results, further research is needed to elucidate the precise mechanisms by which AG, SC, and TSC exert their neuroprotective effects against MG-induced neurotoxicity.

## Data Availability

The dataset presented in the study is available on request from the corresponding author during submission or after publication.
